# Integrated hepatic transcriptional and serum metabolic studies on circulating nutrient metabolism in diurnal laying hens

**DOI:** 10.18632/oncotarget.23032

**Published:** 2017-12-07

**Authors:** Wan Dan, Liu Yi-Lin, Li Guan-Ya, Huang Rui-Lin, Zhang Yi-Ming, Long Ci-Min, Ruan Zheng, Li Lan, Wu Xin, Zhou Xi-Hong, Yin Yu-Long

**Affiliations:** ^1^ Key Laboratory of Agro-Ecological Processes in Subtropical Region, Institute of Subtropical Agriculture, The Chinese Academy of Science, National Engineering Laboratory for Pollution Control and Waste Utilization in Livestock and Poultry Production, Hunan Provincial Engineering Research Center of Healthy Livestock, Scientific Observing and Experimental Station of Animal Nutrition and Feed Science in South-Central, Ministry of Agriculture, Changsha, Hunan 410125, China; ^2^ School of Food Science and Technology, State Laboratory of Food Science and Technology, Nanchang University, Nanchang, Jiangxi 330047, China; ^3^ Animal Nutrition and Human Health Laboratory, School of Life Sciences, Hunan Normal University, Changsha, Hunan 410125, China

**Keywords:** circadian rhythm, laying hens, hepatic transcriptome, circulating nutrients

## Abstract

The aim of the study was to see the diurnal variation of nutrients metabolism and their regulation under the management of large-scaled production. The hepatic transcriptional and serum metabolic studies on circulating nutrient metabolism were investigated in diurnal laying hens. Liver and blood were collected from 36 hens that were slaughtered at 3:30, 7:30, 11:30, 15:30, 19:30, and 23:30 (n = 6), respectively. The serum amino acid, fatty acid and glucose levels, as well as the hepatic transcriptome were analyzed. The results revealed that the circadian clock genes such as Bmal1, Clock, Per1, and Cry2 displayed circadian rhythms in hen livers. The genes related to circulating nutrient transportation, lipogenesis, lipid catabolism, sterol metabolism, and oxidative/anti-oxidative systems also oscillated. However, the nadir of glucose was observed at 7:30 and peaked at 11:30 in the day. Amino acid levels peaked mainly at night, and most amino acids exhibited circadian rhythms based on CircWave analysis. With the exception of undecanoic acid (C11:0), myristoleic acid (C14:1), cis-11, 14-eicosenoic acid (C20:2), and (cis-4, 7, 10, 13, 16, 19-docosahexaenoic acid) C20:3N6 fatty acids, others peaked at 7:30 and 15:30. The results indicated that the hens required more glucose in the early morning. More proteins should be ingested late in the day, since protein catabolism occurred mostly at night. To remove the redundant fats and lipids, fewer should be ingested, especially during the night. All these results would help to design a more accurate nutrition schedule for improving the performance of laying hens in the future.

## INTRODUCTION

The circadian clock programs daily rhythms and coordinates multiple metabolic processes and results in the diurnal oscillation of glucose, amino acids, and fatty acids in wild and clock-/- animals [1–4]. However, the diurnal oscillation of circulating nutrient metabolism is not only regulated by circadian rhythms, but is also affected by the L:D cycle, temperature, and food availability. Once the diurnal oscillation of these metabolites is disrupted, it eventually results in various syndromes, including obesity and glucose tolerance [5], hyperleptinemia, hyperlipidemia, hepatic steatosis, hyperglycemia, and hypoinsulinemia [6].

Circadian rhythms of cells and organismal physiology are controlled by an autoregulatory transcription-translation feedback loop that regulates the expression of rhythmic genes at the transcription level. Studies on the brain, liver, and heart tissues have shown that up to approximately 10% of the transcripts are under circadian clock regulation, in a tissue specific manner [7–11]. In the transcriptional cascade, the CLOCK–BMAL1 complex directly induces the expression of transcription factors that are subsequently responsible for the majority of circadian rhythms. The components of the circadian pacemaker, such as the transcription factor Clock and Per2 gene products, program metabolic processes of cells via RNA transcription periodicity [12]. Although it has been well demonstrated that the circadian clock programs genes involved in metabolism in nocturnal mice, the diurnal variation of the expression of these genes should be studied in diurnal animals, since the expression of circadian pacemakers, i.e., CLOCK and Per, also vary diurnally over 24 h [13–14].

It has been found that changing the daily rhythm of food ingestion also changes the metabolite rhythms. When changing the daily rhythm of methionine ingestion, with 25% of the total methionine ingested in the morning, and 75% at night, the serum total cholesterol (TC), triglyceride (TG), total protein (TP), and calcium of laying hens were significant lower in the early morning [13]. Based on these findings, we focused on the oscillation of circulating nutriments under normal circadian rhythms, L:D cycle, temperature, and food availability. We also hypothesized that changing the ingestion time of nutrients may affect multiple metabolic processes and energy/nutrient storage, and, in food producing animals, may affect production performance.

Therefore, in the current study, diurnal variation of nutrients and genes related to nutrient metabolism were studied in laying hens using hepatic transcriptomic and targeted metabolomic approaches. Using these approaches, we discovered a 24-h rhythm in circulating nutrient metabolism. The diurnal variations of glucose, amino acids, and fatty acids in serum were also investigated. Our results showed that most of the circulating nutrients displayed a time-of-day dependent pattern, and all diurnal variations were highly correlated with the diurnal rhythm of related transcripts.

## RESULTS

### Daily egg production rates

Of all the eggs collected, 72.58 ± 6.33% were collected in the morning (7:30), 23.90 ± 5.75% were collected in the afternoon (12:00), and 3.52 ± 0.94% were collected in the evening (19:30).

### Illumina sequencing and read mapping

RNA-sequencing was used to capture the dynamic temporal changes of liver transcriptomes at 3:30, 7:30, 11:30, 15:30, 19:30, and 23:30 daily (note that hereafter these sampling times will be referred to as C3, C7, C11, C15, C19, and C23 respectively). Six sequencing libraries were prepared and sequenced with the Illumina paired-end method. Approximately 2.5 × 10^7^ raw reads were generated in each group. After removing low quality reads, clean reads were obtained, and > 95% of the clean reads had Phred-like quality scores at the Q20 level (an error probability of 0.01) and GC-contents of approximately 50% (Supplementary Table 1). After RNA sequencing, 74.81–80.24% of the total reads in different samples were mapped to the genome of *G. gallus*; and 73.47–79.02% of the reads in each sample were uniquely mapped to the genome (Supplementary Table 2). Pearson correlations between biological replicates were greater than 0.8, which indicates high reliability of the experiment and rationality of sample selection (Supplementary Table 3).

### Differentially expressed genes

**Figure 1:**
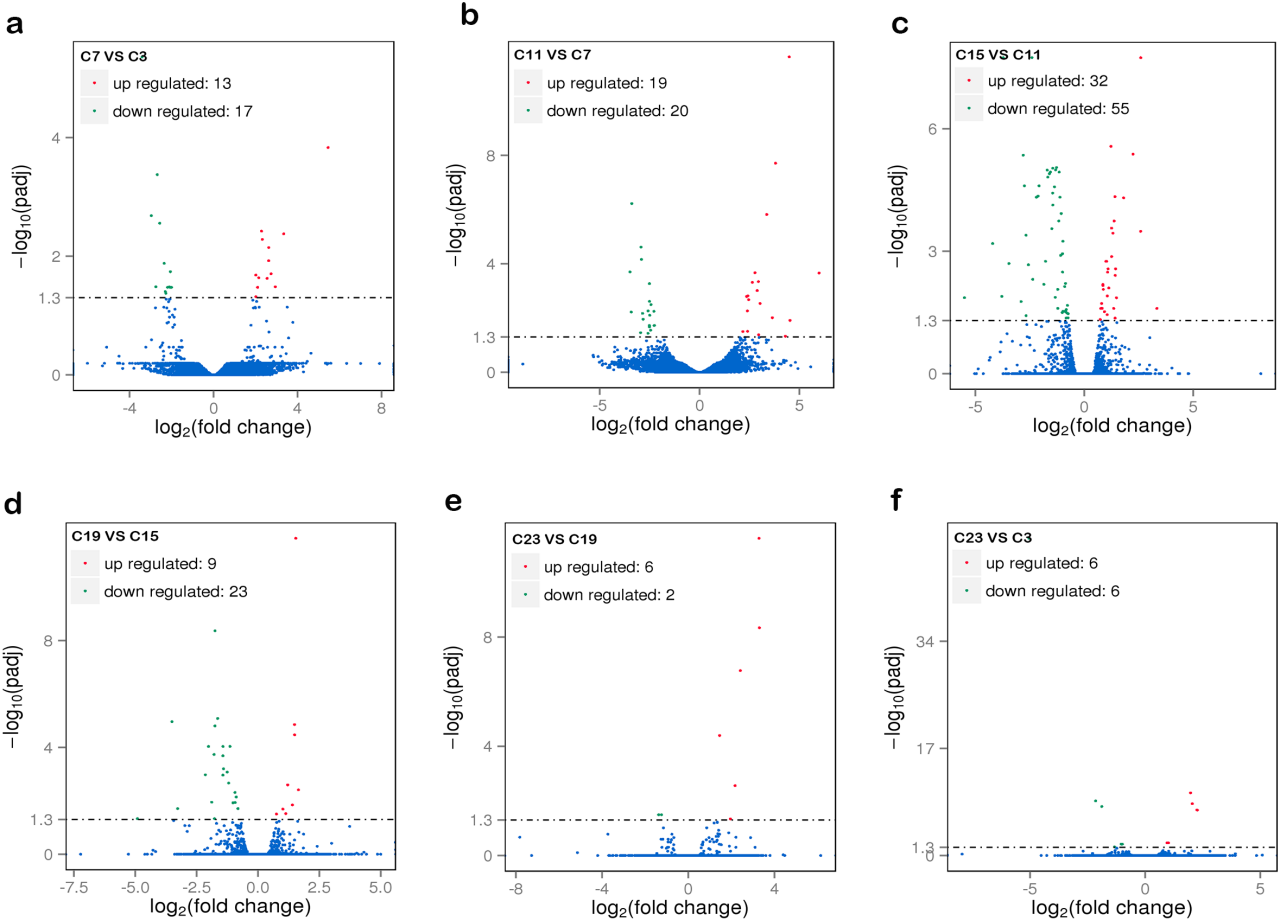
The overall distribution of differentially expressed genes in samples **(a)** Comparison group: C7 and C3; **(b)** comparison group: C11 and C7; **(c)** comparison group: C15 and C11; **(d)** comparison group: C19 and C15; **(e)** comparison group: C23 and C19; **(f)** comparison group: C23 and C3. The horizontal axis shows the change of expression levels of DE genes in different samples; the vertical axis shows the statistical significance of the change of expression levels. Red dots represent genes that are up regulated and green dots represent genes that are down regulated. C3, C7, C11, C15, C19, and C23 refer to samples collected at 03:30, 07:30, 11:30, 15:30, 19:30, and 23:30.

**Figure 2:**
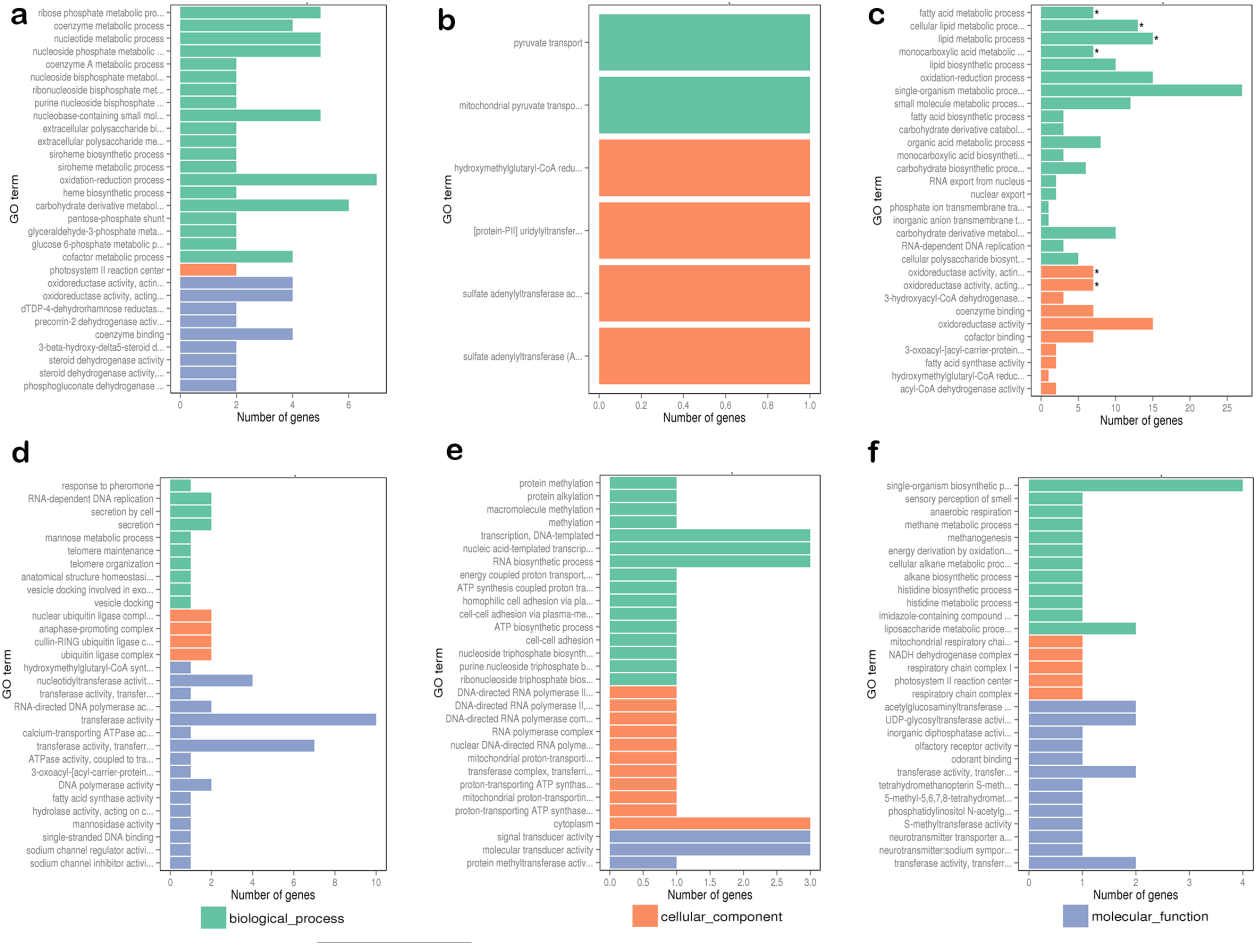
Comparing functional annotations of contigs between different samples **(a)** Comparison group: C7 and C3; **(b)** comparison group: C11 and C7; **(c)** comparison group: C15 and C11; **(d)** comparison group: C19 and C15; **(e)** comparison group: C23 and C19; **(f)** comparison group: C23 and C3. The green bars represent the biological process; orange bars represent the cellular component; purple bars represent the molecular function. C3, C7, C11, C15, C19, and C23 refer to samples collected at 03:30, 07:30, 11:30, 15:30, 19:30, and 23:30.

To better understand the circadian rhythm of genes related to metabolism, the differentially expressed genes between different samples were analyzed. The differentially expressed genes in response to circadian rhythm were clustered as shown in Supplementary Figure 1. The overall distributions of differentially expressed genes of different expression levels in six groups are shown in Figure 1. When comparing the abundance of annotated genes in each group of two sample groups, 419 differentially expressed genes were found, including 74 novel genes. The enriched GO functions were closely related to oxidative metabolism, lipid biosynthesis and metabolism, and heme- or iron-related molecular functions and biological processes, including signal-organism metabolic process, oxidation-reduction processes, oxidoreductase activity, lipid biosynthetic processes, lipid metabolic processes, and iron-ion binding (Figure 2).

### Verification of changes in transcriptomic analysis

**Figure 3:**
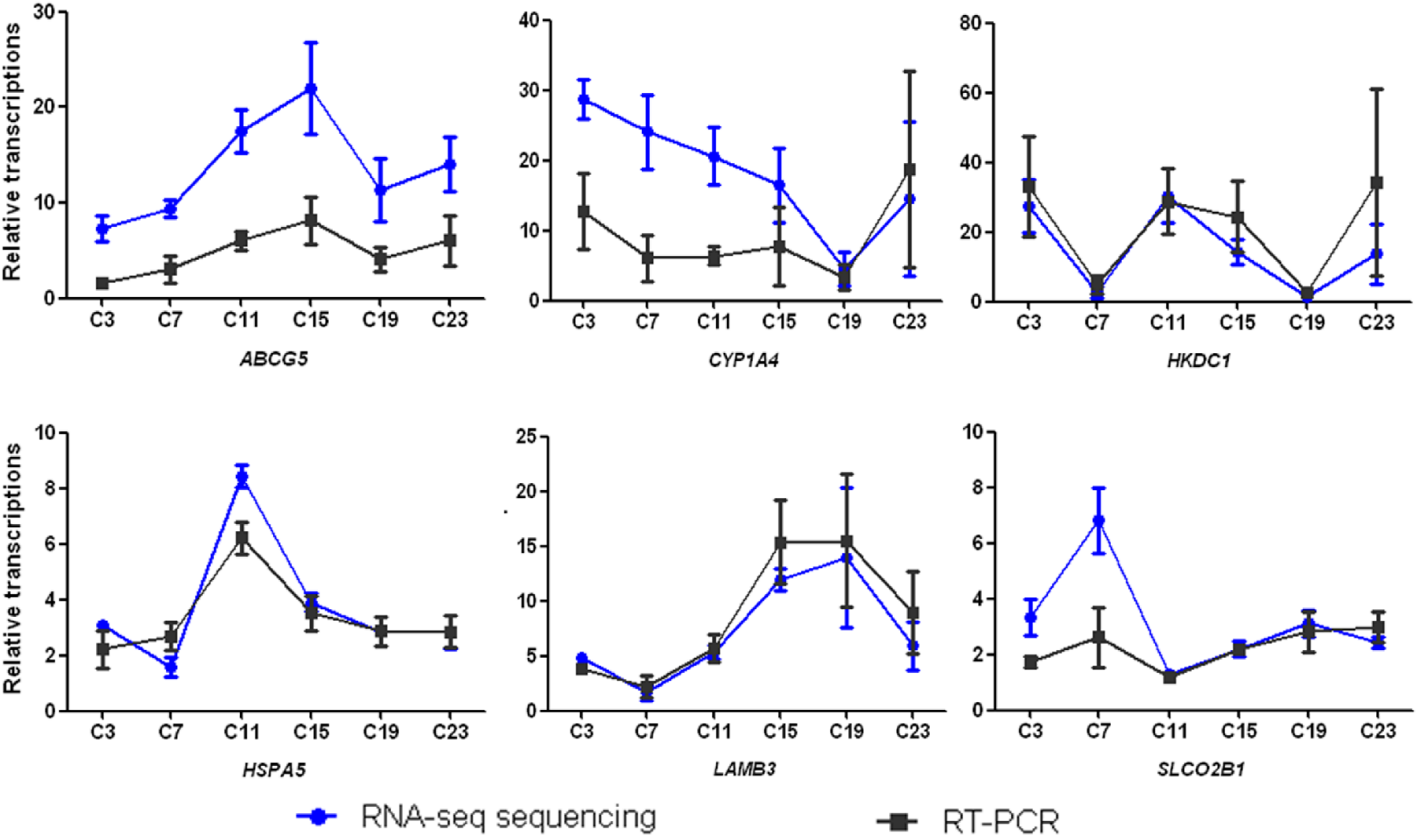
Diurnally regulated transcripts of liver targeted for nutrient metabolism **(a)** Lipid biosynthesis and metabolism; **(b)** protein catabolism and amino acid generation; **(c)** transporters; **(d)** oxidation-reduction metabolism; **(e)** anti-stress response; **(f)** steroid biosynthesis. C3, C7, C11, C15, C19, and C23 refer to samples collected at 03:30, 07:30, 11:30, 15:30, 19:30, and 23:30.

To verify the results from RNA-sequencing, 6 differentially expressed transcriptions namely ATP-binding cassette transporters G5 (ABCG5), cytochrome P450 1A4 enzyme (CYP1A4), Hexokinase Domain Containing 1 (HKDC1), heat shock 70 kDa protein 5 (HSPA5), β3 polypeptide chain of laminin 5 (LAMB3), and organic anion transporting polypeptide 2B1 (SLCO2B1) were randomly selected for RT-PCR analysis. Our results showed that the detection of relative up- and down-regulation of these mRNA levels by RT-PCR was in agreement with the transcriptomics results (Figure 3).

### Diurnal variation of glucose, amino acids, fatty acid metabolism and steroid biosynthesis

Diurnal variation of glucose, amino acids, and fatty acids in serum is shown in Tables 1 and 2. The levels of glucose, amino acids, and most of the fatty acids in serum of laying hens fluctuated in a day. The nadir of glucose was at C7, and glucose reached a peak at C11. Subsequently, glucose remained at a relatively constant concentration in the afternoon and night. However, the highest levels of amino acids were found at night, at C23 or C3. Amino acid levels decreased in the day, and most of the amino acids reached a nadir at C15. With the exception of fatty acids, undecanoic acid (MAG C11:0), myristoleic acid (C14:1), cis-11, 14-eicosenoic acid (C20:2), and (cis-4, 7, 10, 13, 16, 19-docosahexaenoic acid) C20:3N6, which did not vary diurnally, fatty acids had two peak concentrations at C7 and C15. After “CircWave” analysis, the diurnal variation of amino acids including Trp, Ser, Arg, Asp, Thr, Val, Ala, Ile, Pro, Leu, Cys, and Phe exhibited circadian rhythms in the serum of hens.

**Table 1:** Circadian rhythm characteristics of amino acids (μmol/L) in serum^1^

	C3	C7	C11	C15	C19	C23	*P*	SEM	Cosine P	Acrophase (hh:mm)	Mesor	Amplitude
His	22.60 ^a^	21.64 ^a^	23.02 ^a^	15.29^b^	24.09 ^a^	23.12 ^a^	<0.05	0.81	>0.05	-	-	-
Trp	16.10 ^a^	11.42 ^b^	10.47^c^	11.47 ^b^	13.01 ^b^	13.47 ^b^	<0.01	0.43	<0.01	01:00	12.6	2.2
Ser	85.11 ^a^	80.57^a^	77.96^ab^	65.93^b^	83.94^a^	88.01^a^	<0.05	2.05	<0.05	01:40	80.3	8.6
Arg	87.95 ^a^	79.39 ^ac^	68.19 ^c^	52.69 ^b^	75.66 ^ac^	70.90 ^c^	<0.01	2.62	<0.01	03:35	72.7	13.3
Gly	52.56 ^a^	41.33 ^b^	42.20^bc^	42.18 ^bc^	49.48 ^ac^	45.00 ^bc^	<0.05	1.21	>0.05	-	-	-
Asp	16.46 ^a^	10.79 ^bcd^	7.65^cd^	8.25^cd^	13.95 ^ab^	9.82 ^bcd^	<0.05	0.86	<0.05	01:26	11.1	3.1
Glu	39.75 ^a^	28.93 ^bc^	27.83^c^	29.38^bc^	36.81^ab^	30.20 ^bc^	<0.05	1.28	>0.05	-	-	-
Thr	61.00 ^a^	50.48 ^ab^	44.69 ^b^	38.47 ^b^	44.32^b^	42.41 ^b^	<0.05	2.03	<0.01	04:36	47.0	8.8
Val	32.68 ^a^	25.82 ^bc^	24.87 ^bc^	21.21 ^c^	27.57 ^ab^	27.49 ^ab^	<0.05	1.00	<0.01	02:19	26.6	4.3
Ala	50.68^ac^	40.50 ^abg^	42.09 ^abfg^	32.35^b^	54.36 ^c^	51.11 ^acefg^	<0.01	1.94	<0.01	00:05	45.2	8.5
Iie	18.94 ^a^	14.53 ^bc^	13.99 ^bc^	12.08 ^c^	16.06 ^ab^	16.98 ^ab^	<0.01	0.56	<0.01	01:41	15.4	2.9
Pro	53.63^a^	39.14 ^c^	34.80 ^bc^	25.77 ^b^	49.90 ^a^	52.79 ^a^	<0.01	2.21	<0.01	00:57	42.7	13.6
Leu	35.23^a^	29.63 ^c^	30.52 ^c^	24.37^b^	34.04 ^ac^	33.65^ac^	<0.01	0.91	<0.01	01:20	31.2	4.1
Cys	9.60 ^a^	6.56 ^abce^	6.00 ^cbde^	2.50 ^df^	3.80 ^bef^	3.47^bf^	<0.01	0.62	<0.01	05:40	5.3	2.9
Phe	22.64^ac^	17.81^b^	17.44 ^b^	17.34 ^b^	21.89^cd^	19.18^bd^	<0.01	0.54	<0.01	00:07	19.3	2.2
Tyr	38.14^a^	35.96^a^	35.14 ^a^	35.93^a^	38.99^a^	28.45^b^	<0.05	0.99	>0.05	-	-	-

^1^ The amino acids that of p >0.05 after ANOVA analysis were not involved.

(–) depicts no significance in cosinor waveform characteristics.

**Table 2:** Diurnal variation of glucose and fatty acids in serum

	C3	C7	C11	C15	C19	C23	*P*	SEM	Cosine P
**Glucose (mmol/L)**									
Glu	12.89 ^ac^	11.45 ^bc^	13.41^a^	13.19 ^ac^	12.03 ^bc^	12.20^abc^	0.02	0.21	0.35
**Fatty acids (mg/L)**									
C11:0	2.27	2.24	2.45	2.36	2.51	2.06	0.96	0.14	0.77
C14:0	68.93 ^a^	104.80 ^b^	59.70^a^	64.83^a^	81.63 ^ab^	72.92 ^a^	0.07	4.46	0.57
C14:1	63.71	70.81	56.28	65.92	61.69	59.48	0.61	2.31	0.86
C15:0	9.90 ^a^	14.54 ^b^	8.51 ^a^	9.94 ^a^	12.09 ^ab^	10.82 ^ab^	0.08	0.60	0.73
C16:0	5798.60 ^a^	8648.73 ^b^	4731.54^a^	5340.59 ^a^	6851.06 ^ab^	5962.55 ^a^	0.06	379.06	0.56
C16:1	551.93^a^	1054.67 ^b^	354.66^c^	492.24^a^	789.33^ab^	580.44 ^abc^	0.01	59.10	0.56
C17:0	36.09 ^ab^	50.07 ^b^	31.69 ^a^	33.26^a^	40.49^ab^	37.05^ab^	0.30	2.29	0.63
C18:0	2821.59 ^ab^	3455.79 ^b^	2556.58 ^a^	2691.48 ^a^	3172.83 ^ab^	2732.58 ^ab^	0.16	106.07	0.81
C18:1N9C	5623.24 ^a^	9402.78 ^b^	4292.64^a^	4644.44 ^a^	7394.63 ^ab^	5976.66 ^a^	0.06	522.71	0.53
C18:1N9T	290.92^a^	503.92^b^	237.31^a^	248.66^a^	374.35^ab^	316.91^a^	0.02	24.41	0.45
C18:2N6C	2176.44 ^a^	3439.05 ^b^	1515.19^c^	1917.63^a^	2687.94 ^ab^	2385.32^abc^	0.04	176.79	0.42
C20:0	16.39	19.14	16	17.61	18.31	14.94	0.55	0.70	0.75
C20:1	17.96 ^a^	30.88 ^b^	10.77^c^	11.48^a^	22.19 ^ab^	19.58^ac^	0.01	1.81	0.18
C20:3N3	21.33^a^	35.88^b^	16.34^a^	19.00^a^	25.69 ^ab^	23.17^ab^	0.08	1.92	0.49
C20:4N6	416.33^a^	768.39^b^	343.93 ^a^	383.70^a^	529.93^ab^	467.67^a^	0.04	39.93	0.51
C22:6N3	70.86	149.47	59.49	60.32	153.41	87.57	0.31	15.72	0.86

### Diurnal variation in transcripts in hens’ liver

**Figure 4:**
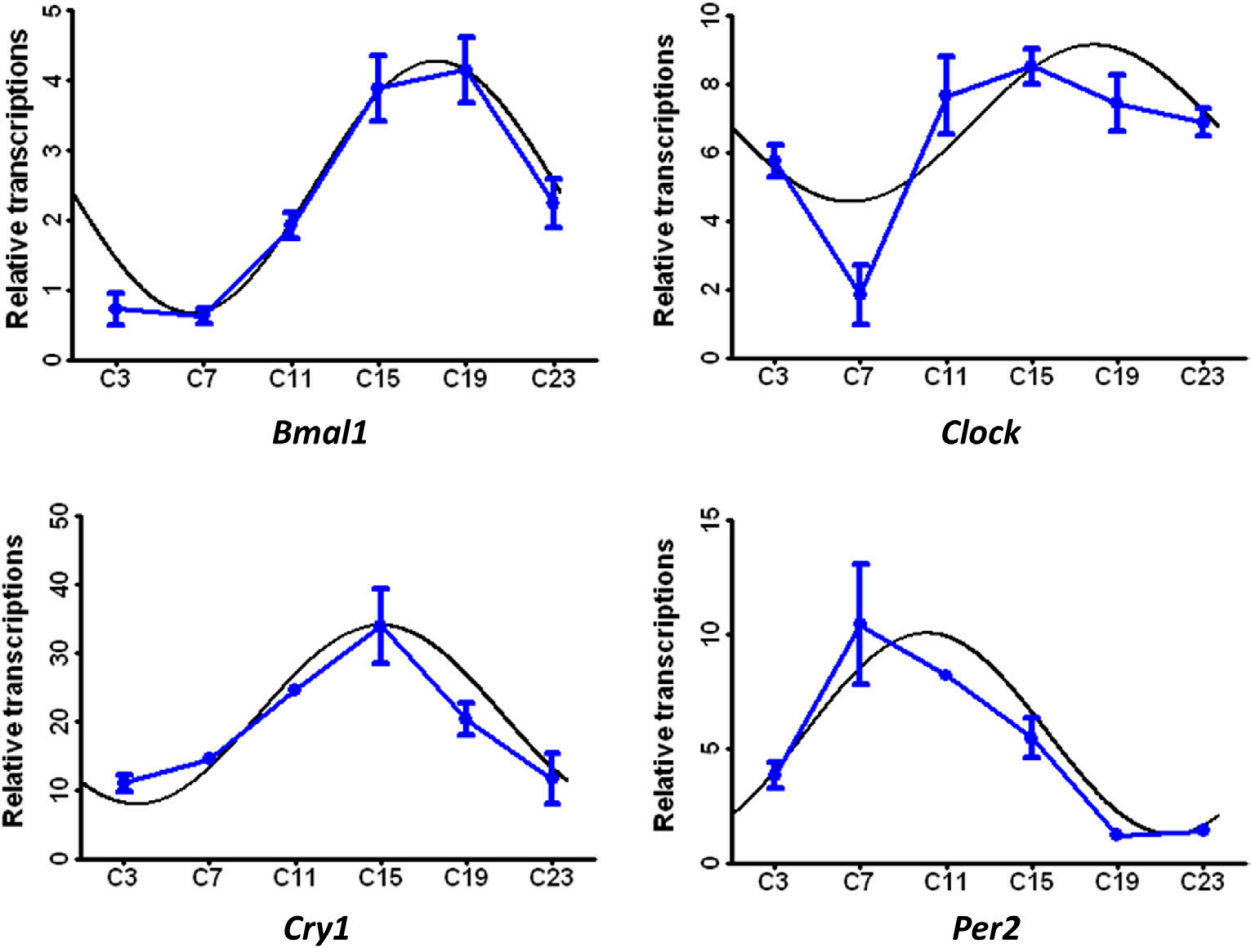
Confirmation of transcriptomics quantification by qRT-PCR **(a)** *ABCG5*, ATP-binding cassette transporters G5; **(b)** *CYP1A4*, cytochrome P450 1A4 enzyme; **(c)** *HKDC1*, Hexokinase Domain Containing 1; **(d)** *HSPA5*, heat shock 70 kDa protein 5; **(e)** *LAMB3*, β3 polypeptide chain of laminin 5 and **(f)** *SLCO2B1*, organic anion transporting polypeptide 2B1. C3, C7, C11, C15, C19, and C23 refer to samples collected at 03:30, 07:30, 11:30, 15:30, 19:30, and 23:30.

**Figure 5:**
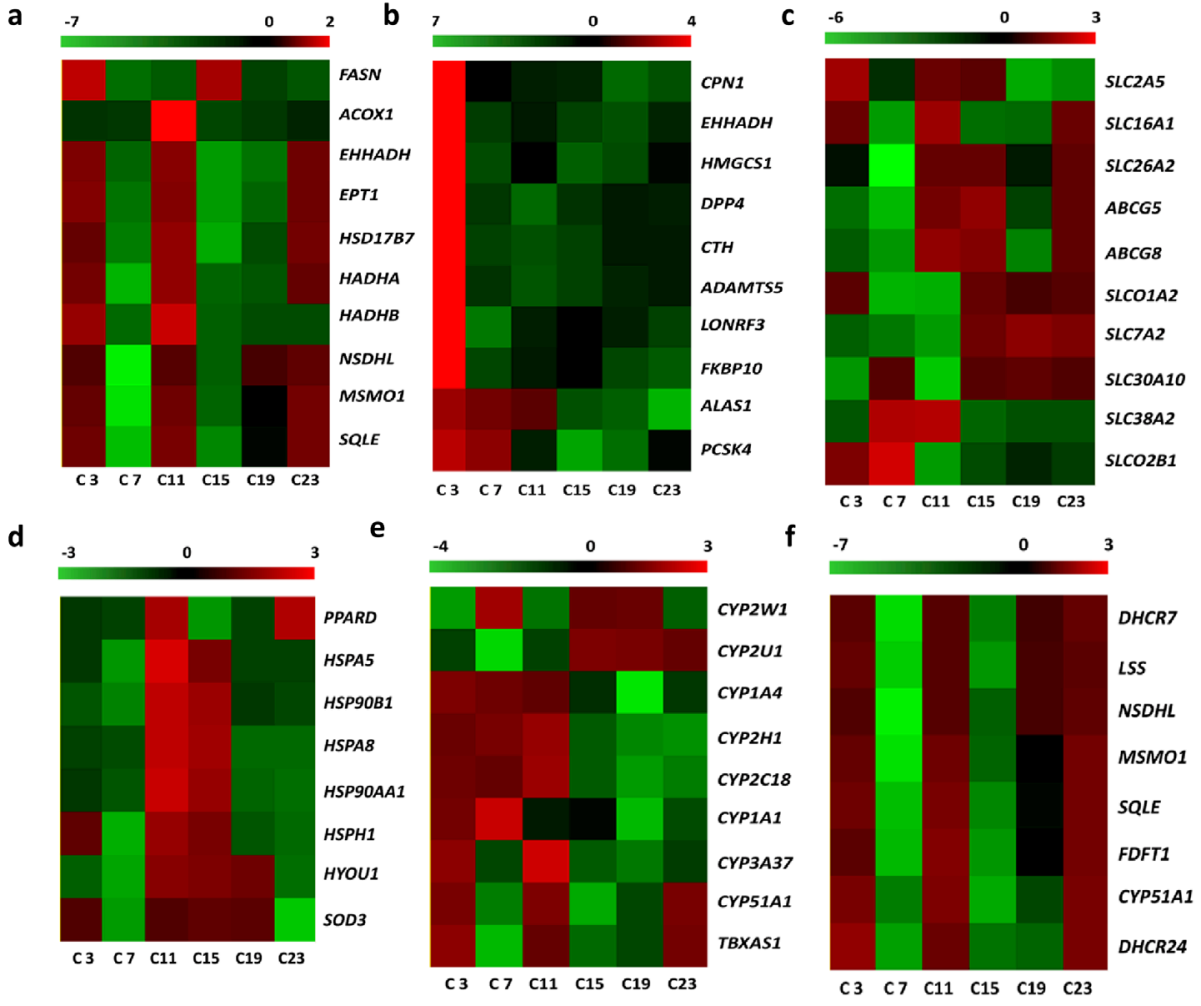
Diurnally regulated transcripts of liver targeted for nutrient metabolism **(a)** protein catabolism; **(b)** lipid and fatty acid metabolism; **(c)** transporters; **(d)** oxidative stress and anti-stress responses; **(e)** endogenic sterol and xenobiotic metabolism; **(f)** steroid biosynthesis C3, C7, C11, C15, C19, and C23 refer to samples collected at 03:30, 07:30, 11:30, 15:30, 19:30, and 23:30.

The diurnally varied genes related to circadian rhythms are shown in Figure 4. After CircWave analysis, the genes, including Bmal1, Clock, Per1 and Cry2, exhibited circadian rhythms in the livers of hens. Meanwhile, the transcripts of enzymes involved in protein catabolism, i.e., peptidase and aminotransferase peaked at C3 (Figure 5a), However, the transcripts that were encoded by the genes involved in lipid and fatty acid metabolism had two nadirs, in contrast to the free fatty acid levels in serum (Figure 5b). Moreover, the transporters related to nutrient transport in solute carrier family included SLC2A5, SLC16A1, SLC26A2, SLC7A2, SLC38A2, and SLC30A10 (Figure 5c). These genes are involved in glucose/oligosaccharides, monocarboxylic acid, sulfate anion, cationic amino acid, and sodium-coupled neutral amino acid transportation, respectively.

However, transcripts related to oxidative stress and anti-stress responses were also found to be differentially expressed. The molecular chaperones, including heat shock protein *Hsp A5*, *Hsp A8*, *Hsp90 AA1*, *Hsp 90B1*, *Hsp H1*, and *HYOU1* (*Hsp12A*) are diurnally expressed in response to daily stresses. The peroxisome proliferator-activated receptor delta (*PPARD*) and Cu-Zn superoxide dismutase (*SOD3*) were also diurnally expressed (Figure 5d). The *ABCG5*, *ABCG8*, *SLC01A2*, and *SLCO2B1* transporters, which are involved in drug and xenobiotic transportation, also displayed time-of-day dependent rhythms. In addition, the CYP oxidases that targeted endogenic sterol and xenobiotic oxidation and reduction also displayed diurnal variations (Figure 5e).

KEGG pathway analysis of differentially expressed genes showed that only a small number of genes were annotated to the KEGG pathways and most of these were related to steroid biosynthesis. Therefore, the transcripts related to dynamic synthesis of steroids were clustered in Figure 3f. The enriched genes displayed time-of–day rhythms. One of the steroids, cholesterol, also displayed time-of-day rhythms.

## DISCUSSION

The 24-h rhythms of targeted metabolites, including glucose, fatty acids, and amino acids were investigated in laying hens during egg production. Except for fatty acids C11.0, C14.1, C20.2, and C20.6N3, all metabolites investigated displayed time-of-day rhythms. The variation of these metabolites was closely related to the genes for transportation, lipogenesis, lipid catabolism, sterol metabolism, as well as oxidative/anti-oxidative systems in the liver. These findings are partially consistent with results found in nocturnal mice, where expression of metabolic enzymes, transport systems involved in cholesterol metabolism, amino acid regulation, drug and toxin metabolism, the citric acid cycle, and glycogen and glucose metabolism varied diurnally in response to *Clock* control, but the trends of individual metabolites were different [2, 7].

The circadian clock has been reported to regulate glycometabolism, and glucose has been shown to phase-shift the clock in peripheral tissues in various animals [15]. In laying hens, the plasma glucose concentrations peaked at C11 and reached a nadir at C7. These trends were similar to those found in human adolescents, where glucose significantly increased starting at 5:30 [16–17]. However, glucose reached a peak in the middle of the night in nocturnal mice [18]. In diurnal horses and sheep, plasma glucose concentrations peaked around the middle of the night and at dusk [19]. The fluctuation of glucose concentrations in plasma mainly results from the coordinated regulation of glucose input (food intake, hepatic glucose production) and its utilization (uptake by skeletal and cardiac muscles, and adipose tissues). Since energy requirements of organisms fluctuate temporally throughout the day, diurnal oscillations in glucose metabolism are partially owing to daily changes in sleep/wake cycles and glucose utilization [20]. In our study, the glucose concentration in blood reached its nadir in the morning after consumption, and increased immediately after ingestion, suggesting the diurnal variation of glucose is related to feeding/fasting cycles.

The effects of the circadian clock on proteometabolism have not been extensively studied in diurnal animals. In the current study, we found that most of the amino acids in plasma and the transcripts related to protein catabolism peaked at night in hens. Moreover, it has been reported that the transcription of amino acid transporters, including *SLC1A2*, *SLC6A20*, *SLC7A1*, and *SLC6A14* was higher at 23:30, 3:30, and 7:30 than at 11:30, 15:30, and 19:30 in piglets [21], suggesting amino acid uptake also peaked at night. Similar results were found in mice where amino acid metabolism-related biochemicals peaked at night [2]. Together, both the diurnal and nocturnal study animals displayed similar rhythms in proteometabolism. The regulation of amino acid metabolism by the circadian clock is not simply an indirect effect of its regulation of glucose metabolism [2], but also directly affected by the diurnal variation of protein catabolism and amino acid uptake.

For lipid metabolism, higher levels of plasma free fatty acids (FFA) were found at night in humans because of enhanced lipolytic activity [22–23]. However, FFA levels are lower at night and increase in the early morning in goats and horses [24–25]. In rodents, the long chain fatty acids margarate (17:0), 10-heptadecenoate (17:1n7), and oleate (18:1n9) all peaked at 9:30 [2]. In pigs, most of the long chain fatty acids peaked at night [14]. However, in the current study, we found that long chain fatty acids in the serum of hens fluctuated over 24 h, and most of the long chain fatty acids had two peaks at 7:30 and 19:30. Furthermore, this fluctuation coincides with the variation of transcripts related to lipid synthesis, lipid metabolism, and fatty acid metabolism, including *ACOX1*, *EHHADH*, *SQLE*, *HADHA*, *HADHB*, *EPT1*, *HSD17B7*, *FASN*, *NSDHL*, and *MSMO1*. However, the genes involved in lipid synthesis that were previously reported to vary diurnally in pigs and mice [14, 26–27], such as apolipoprotein B (apoB), fatty acid binding protein (FABP), delta 5-desaturase (FASD1), delta 6-desaturase (FASD2), fatty acid elongase 5 (ELOVL5), and fatty acid elongase 2 (ELOVL2), were not found in hens.

Collectively, our results showed that the de novo synthesis of steroid hormones, and the metabolism of glucose, proteins, lipids, as well as oxidative and anti-oxidative enzymes displayed time-of-day rhythms. Since nutritional challenges could reprogram the circadian clock, these findings are of great importance in animal precision nutrition, and prevention of nutritional and metabolic diseases related to the circadian clock.

## MATERIALS AND METHODS

### Experimental design and sample collection

A total of 60 brown Hy-line laying hens were selected at 41 wk (Xingjia Inc., Changsha, China) and housed individually in 39 × 35 × 38 cm wire cages. Hens were kept under a 16L:8D cycle from 06:00 to 22:00 with *ad libitum* access to corn-soybean-based diets (NRC, 1994) [28] (Supplementary Table 4) and drinking water for 70 d to acquire the same biological rhythm. During these days, eggs were collected and counted daily at 07:30, 12:00, and 19:30 to calculate diurnal egg-laying rates. Subsequently, blood samples (n = 6 per sampling time point) of the hens were collected intravenously at 4-h intervals in a daily cycle starting at 03:30, and then livers were harvested, rinsed in PBS, and snap frozen in liquid nitrogen for RNA sequencing. Particularly, the heads of the chickens that slaughtered at 3:30 and 23:30 were with lightproof bags to avoid light exposure.

### Transcriptomic study

Liver samples of 36 hens that were slaughtered at 3:30, 7:30, 11:30, 15:30, 19:30, and 23:30 (n = 6 at each sampling time) were used for total RNA isolation. After quantification, the six samples in each group were pooled into three biological replicates and submitted for DGE experiments by Novogene Bioinformatics Technology Co. Ltd (Beijing) on an Illumina Hiseq platform, and 125/150 bp paired-end reads were generated. Total reads were mapped to the *Gallus gallus* genome in ensemble (http://www.ensembl.org/Gallus_gallus/Info/Index) using Bowtie v2.2.3 and TopHat v2.0.12 software. HTSeq v0.6.1 was used to count the read numbers mapped to each gene based on the length of the gene and read count mapped to this gene. Differential expression analysis of two groups was performed using the DESeq R package (1.18.0). The resulting P-values were adjusted using Benjamini and Hochberg’s approach for controlling the false discovery rate. Genes with log2 fold change >1 and adjusted P-value < 0.05 found by DESeq were assigned as differentially expressed.

### Real-time polymerase chain reaction (RT-PCR)

Quantitative real-time polymerase chain reaction (RT-PCR) was performed to validate the results of transcriptome sequencing and determine the expression levels of the selected genes in livers in different sampling periods. Fold differences in expression levels were calculated using the 2^-ΔΔCt^method. Primers and amplicon sizes are listed in Supplementary Table 5.

### Glucose, amino acids, and fatty acids analyses

Serum glucose was assessed using a discrete type of auto analyzer, the Hitachi 7150 (Hitachi Medical, Tokyo, Japan). Serum free amino acids were measured on an Automatic Amino Acid Analyzer (Hitach L-8800) as previously described. PUFA contents were analyzed according to previously reported methods by GC-MS, using 19:0 methyl ester as an internal standard [14].

### Statistical analysis

Statistical analysis was performed using SPSS 17.0 for Windows (SPSS Inc., Chicago, IL). All results are expressed as Mean values. The differences among treatments were evaluated using one-way analysis of variance (ANOVA), considering time point as a fixed effect and hen ID as a randomized effect. Data are presented as least squares means ± SEM. Data were also analyzed by “CircWave” software (v. 1.4, courtesy of R. Hut; http://www.euclock.org) to confirm the diurnal rhythmicity. Probability values < 0.05 were considered statistically significant.

## SUPPLEMENTARY MATERIALS FIGURE AND TABLES



## Abbreviations

PUFA: long chain polyunsaturated FA
FFA: free fatty acids
CYPs: cytochrome oxidases
HSP: heat shock proteins
TC: total cholesterol
TG: triglyceride
TP: total protein
RT-PCR: Quantitative real-time polymerase chain reaction
HPA: hypothalamic-pituitary-gonadal
PPARD: peroxisome proliferator-activated receptor delta
SOD3: Cu-Zn superoxide dismutase
ABCG5: ATP-binding cassette transporters G5
CYP1A4: cytochrome P450 1A4 enzyme
HKDC1: Hexokinase Domain Containing 1
HSPA5: heat shock 70 kDa protein 5
LAMB3: β3 polypeptide chain of laminin 5
SLCO2B1: organic anion transporting polypeptide 2B1
