# Supplementary material for: Integrated hepatic transcriptional and serum metabolic studies on circulating nutrient metabolism in diurnal laying hens
Source: Oncotarget. 2017 Dec 7;8(69):113885–94. doi: 10.18632/oncotarget.23032 (PMC5768371; doi:10.18632/oncotarget.23032)
Supplement: Supplementary file 1 [file oncotarget-08-113885-s001.pdf]

Integrated hepatic transcriptional and serum metabolic studies on circulating nutrient metabolism in diurnal laying hens

SUPPLEMENTARY MATERIALS

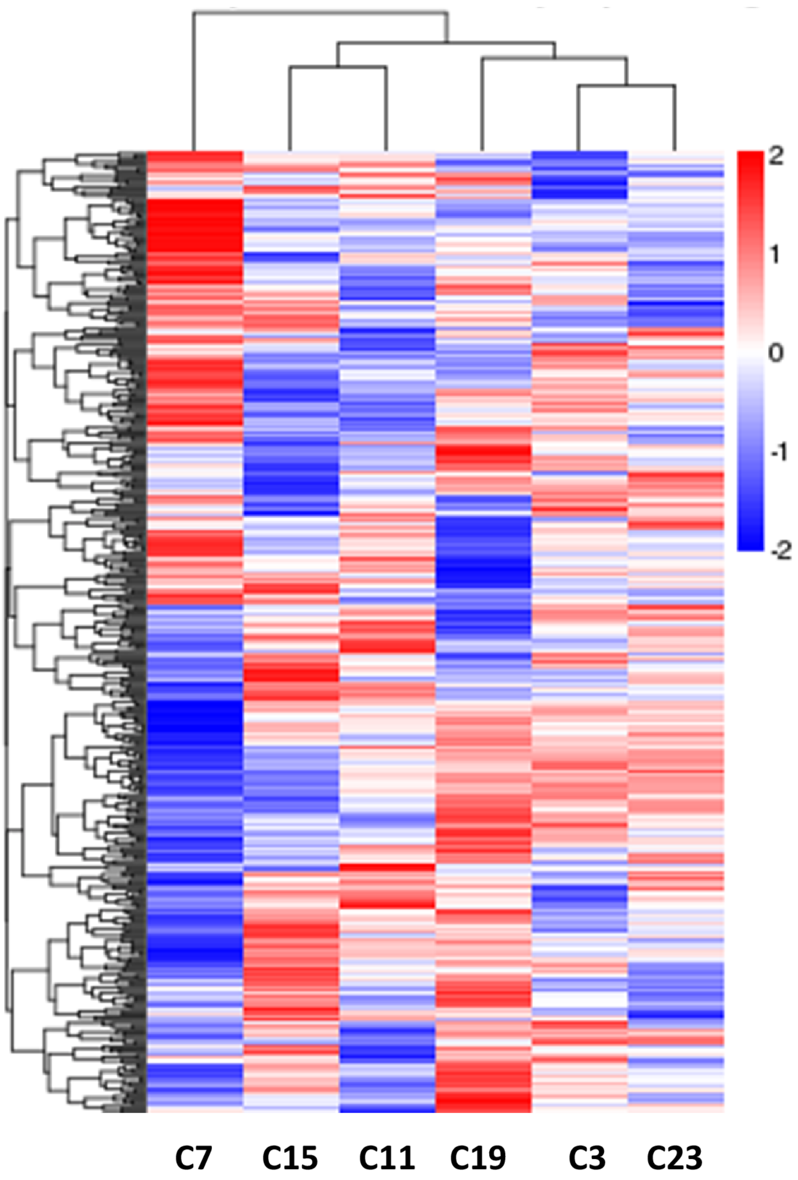

Supplementary Figure 1: Cluster of differentially expressed genes. C3, C7, C11, C15, C19, and C23 refer to samples collected at 03:30, 07:30, 11:30, 15:30, 19:30, and 23:30.

Supplementary Table 1: Quality of sequencing

Sample name	Raw reads	Clean reads	clean bases	Error rate (%)	Q20 (%)[a]	Q30 (%)[b]	GC content (%)
**C3_1**	24921404	23998326	3.60 G	0.02	96.31	91.05	48.81
**C3_2**	27497480	26462394	3.97 G	0.02	96.21	90.83	48.81
**C3_3**	29823102	28581466	4.29 G	0.02	95.87	90.19	49.26
**C7_1**	25418996	20848382	3.13 G	0.01	98.04	93.62	50.3
**C7_2**	30977672	24210164	3.63 G	0.02	97.71	92.9	50.63
**C7_3**	24732804	23844948	3.58 G	0.02	95.94	90.18	48.81
**C11_1**	26690650	25571286	3.84 G	0.02	95.59	89.49	48.58
**C11_2**	24149710	23254438	3.49 G	0.02	95.85	90.01	49.06
**C11_3**	23673776	22884552	3.43 G	0.02	96.07	90.39	48.66
**C15_1**	23710246	22936280	3.44 G	0.02	96.21	90.67	48.63
**C15_2**	26550646	25588658	3.84 G	0.02	95.98	90.24	48.23
**C15_3**	23738982	22966164	3.44 G	0.02	96.29	90.87	48.1
**C19_1**	22439426	21618630	3.24 G	0.02	95.96	90.23	48.15
**C19_2**	21688780	20786394	3.12 G	0.02	95.78	89.86	49.69
**C19_3**	21310744	20531316	3.08 G	0.02	96.02	90.33	48.74
**C23_1**	25801706	24911084	3.74 G	0.02	96.12	90.54	48.66
**C23_2**	24825922	23992398	3.60 G	0.02	96.33	90.95	48.74
**C23_3**	24096356	23169244	3.48 G	0.02	96.24	90.75	48.4

[a] percentage is the proportion of nucleotides with a quality value >20 in reads.
[b] percentage is the proportion of nucleotides with a quality value >30 in reads.

Supplementary Table 2: The percentages of reads and expressed genes mapping to the reference genome

Sample	Total mapped reads		Uniquely mapped reads		Exon (%)	Intron (%)	Intergenic (%)	Num. of expressed genes[a]	Num. of highly expressed genes [b]
	Num.	%	Num.	%					
**C3_1**	19257221	80.24	18963414	79.02	83.6	4.2	12.1	14179	861(5.06%)
**C3_2**	21100912	79.74	20723407	78.31	84.6	3.8	11.7	14126	930(5.46%)
**C3_3**	22365610	78.25	22019662	77.04	82.9	4.3	12.6	14501	947(5.56%)
**C7_1**	15596547	74.81	15317101	73.47	76.7	5.8	17.5	14032	952(5.59%)
**C7_2**	18212614	75.23	17835818	73.67	78.4	5.3	16.3	14275	995(5.85%)
**C7_3**	18872177	79.15	18536897	77.74	83.3	4.5	12.2	14215	893(5.25%)
**C11_1**	20216379	79.06	19890200	77.78	85.1	4.1	10.8	14046	875(5.14%)
**C11_2**	18353617	78.93	18030428	77.54	83.6	4.5	11.7	14156	919(5.40%)
**C11_3**	18222355	79.63	17947752	78.43	83.3	4	12.7	14177	925(5.43%)
**C15_1**	18117219	78.99	17797914	77.6	81.9	4.9	13.2	14054	891(5.23%)
**C15_2**	20171303	78.83	19839509	77.53	82.1	4.5	13.4	14270	906(5.32%)
**C15_3**	18292359	79.65	18010998	78.42	82	5	13	14040	858(5.04%)
**C19_1**	17101141	79.1	16887401	78.12	84.7	4.8	10.5	14073	875(5.14%)
**C19_2**	15804572	76.03	15491006	74.52	82.8	4.8	12.4	14068	926(5.44%)
**C19_3**	16223786	79.02	15939786	77.64	84.3	4.4	11.3	13868	833(4.89%)
**C23_1**	19309633	77.51	19019949	76.35	81.1	5.3	13.6	14280	921(5.41%)
**C23_2**	18825714	78.47	18515976	77.17	83	4.4	12.7	14146	893(5.25%)
**C23_3**	18058623	77.94	17794053	76.8	81.9	4.8	13.3	14185	862(5.06%)

[a] FPKM > 0.
[b] FPKM > 60.

Supplementary Table 3: Pearson correlations between samples

R^2	C3_1	C3_2	C3_3	C7_1	C7_2	C7_3	C11_1	C11_2	C11_3	C15_1	C15_2	C15_3	C19_1	C19_2	C19_3	C23_1	C23_2	C23_3
**C3_1**	1	0.968	0.96	0.812	0.834	0.962	0.965	0.961	0.964	0.952	0.961	0.964	0.956	0.96	0.959	0.964	0.964	0.966
**C3_2**	0.968	1	0.965	0.791	0.814	0.965	0.965	0.959	0.963	0.96	0.969	0.966	0.966	0.963	0.96	0.964	0.968	0.967
**C3_3**	0.96	0.965	1	0.783	0.806	0.956	0.948	0.946	0.953	0.946	0.957	0.957	0.957	0.959	0.952	0.967	0.96	0.962
**C7_1**	0.812	0.791	0.783	1	0.964	0.804	0.778	0.798	0.78	0.788	0.793	0.798	0.79	0.794	0.79	0.788	0.795	0.788
**C7_2**	0.834	0.814	0.806	0.964	1	0.822	0.803	0.82	0.802	0.815	0.816	0.817	0.81	0.815	0.808	0.807	0.815	0.81
**C7_3**	0.962	0.965	0.956	0.804	0.822	1	0.947	0.952	0.953	0.949	0.958	0.962	0.959	0.957	0.963	0.962	0.96	0.963
**C11_1**	0.965	0.965	0.948	0.778	0.803	0.947	1	0.964	0.968	0.958	0.965	0.964	0.959	0.957	0.952	0.954	0.964	0.961
**C11_2**	0.961	0.959	0.946	0.798	0.82	0.952	0.964	1	0.96	0.961	0.963	0.962	0.955	0.959	0.952	0.953	0.961	0.954
**C11_3**	0.964	0.963	0.953	0.78	0.802	0.953	0.968	0.96	1	0.953	0.964	0.962	0.958	0.954	0.953	0.961	0.963	0.961
**C15_1**	0.952	0.96	0.946	0.788	0.815	0.949	0.958	0.961	0.953	1	0.971	0.968	0.962	0.96	0.956	0.948	0.962	0.957
**C15_2**	0.961	0.969	0.957	0.793	0.816	0.958	0.965	0.963	0.964	0.971	1	0.974	0.969	0.966	0.962	0.962	0.968	0.966
**C15_3**	0.964	0.966	0.957	0.798	0.817	0.962	0.964	0.962	0.962	0.968	0.974	1	0.966	0.964	0.965	0.962	0.966	0.965
**C19_1**	0.956	0.966	0.957	0.79	0.81	0.959	0.959	0.955	0.958	0.962	0.969	0.966	1	0.965	0.963	0.964	0.97	0.966
**C19_2**	0.96	0.963	0.959	0.794	0.815	0.957	0.957	0.959	0.954	0.96	0.966	0.964	0.965	1	0.963	0.958	0.968	0.963
**C19_3**	0.959	0.96	0.952	0.79	0.808	0.963	0.952	0.952	0.953	0.956	0.962	0.965	0.963	0.963	1	0.959	0.965	0.963
**C23_1**	0.964	0.964	0.967	0.788	0.807	0.962	0.954	0.953	0.961	0.948	0.962	0.962	0.964	0.958	0.959	1	0.966	0.969
**C23_2**	0.964	0.968	0.96	0.795	0.815	0.96	0.964	0.961	0.963	0.962	0.968	0.966	0.97	0.968	0.965	0.966	1	0.968
**C23_3**	0.966	0.967	0.962	0.788	0.81	0.963	0.961	0.954	0.961	0.957	0.966	0.965	0.966	0.963	0.963	0.969	0.968	1

Supplementary Table 4: The ingredient composition and the nutrient content of the diet

Ingredients (%)		Nutrient and energy content (%)[2]	
Corn	60.00	ME (kcal/kg)	2740.20
Soybean meal	22.00	Crude protein	15.24
Wheat bran	6.00	Crude fat	2.75
Limestone	7.98	Crude fiber	2.64
Dicalcium phosphate	1.31	Ash	11.85
NaCl	0.30	Ca	3.40
Zeolite Powder	2.09	Available phosphorus	0.34
DL-Methionine	0.06		
premix[1]	0.26		
Total	100.00		

[1] Supplied per kilogram of diet: vitamin A, 12,000 IU; vitamin D3, 3,000 IU; vitamin E, 30 mg; vitamin K3, 6 mg; vitamin B1, 3 mg; vitamin B2, 9 mg; vitamin B6, 6 mg; vitamin B12, 0.03 mg; D-biotin, 0.15 mg; D-Pantothenic acid, 18 mg; 1.5 mg of Folic acid acid; 6 mg of nicotinamide; 18.15 mg of ethoxyquin; choline chloride, 50 mg; phytase, 10 mg; ubiquitin calcium, 0.004 mg; Cu, 5.12 mg as $CuSO_4.5H_2O$; Fe, 72 mg as $FeSO_4.7H_2O$; Zn, 56 mg as $ZnSO_4.H_2O$; Mn, 84.8 mg as $MnSO_4.H_2O$; I, 0.64 mg as KI; Se, 0.24 mg as Na_2SeO_4 ; Co, 0.32 mg as $CoCO_3$.

[2] Calculated values.

Supplementary Table 5: Primers used for qRT-PCR analysis

Gene name	Primer sequences (5'-3')	Product length	Amplified region (bp)
Actin	F:TTACTCGCCTCTGTGAAGGC	228	1477-1704
	R:TCCTAGACTGTGGGGGACTG		
SLCO2B1	F:GTAAAAGCACTGAATGGCCCC	269	169-437
	R:AGCGCTTCTCGATGGTTGAG		
ABCG5	F:TTTCTGGAGGTGAGAGGCGT	184	743-926
	R:GAGCGAGGCTGATGAATGGT		
CYP1A4	F:AAGTTGGCATCATGCAGGGA	198	1795-1992
	R:GCAGCCAGAAGTGTTCTCCT		
HKDC1	F:CTAGCAGCCATACTGACCCG	277	1679-1955
	R:CCAGGGTTAGCACGAAAGGT		
HSPA5	F:CGTGGTGTCCCACAGATTGA	139	1511-1649
	R:TCTCCTCTGGTGTTAGCCGA		
LAMB3	F:CTGCACACTTCGTAGCCGTA	188	268-455
	R:GGCTGCCTCACTTTACTGAC		

Note: The primers were manufactured by Sangon Biotech Co. Ltd. (Shanghai, PR China).
